# Csm4-Dependent Telomere Movement on Nuclear Envelope Promotes Meiotic Recombination

**DOI:** 10.1371/journal.pgen.1000196

**Published:** 2008-09-26

**Authors:** Hiromichi Kosaka, Miki Shinohara, Akira Shinohara

**Affiliations:** Institute for Protein Research, Graduate School of Science, Osaka University, Suita, Osaka, Japan; National Cancer Institute, United States of America

## Abstract

During meiotic prophase, chromosomes display rapid movement, and their telomeres attach to the nuclear envelope and cluster to form a “chromosomal bouquet.” Little is known about the roles of the chromosome movement and telomere clustering in this phase. In budding yeast, telomere clustering is promoted by a meiosis-specific, telomere-binding protein, Ndj1. Here, we show that a meiosis-specific protein, Csm4, which forms a complex with Ndj1, facilitates bouquet formation. In the absence of Csm4, Ndj1-bound telomeres tether to nuclear envelopes but do not cluster, suggesting that telomere clustering in the meiotic prophase consists of at least two distinct steps: Ndj1-dependent tethering to the nuclear envelope and Csm4-dependent clustering/movement. Similar to Ndj1, Csm4 is required for several distinct steps during meiotic recombination. Our results suggest that Csm4 promotes efficient second-end capture of a double-strand break following a homology search, as well as resolution of the double-Holliday junction during crossover formation. We propose that chromosome movement and associated telomere dynamics at the nuclear envelope promotes the completion of key biochemical steps during meiotic recombination.

## Author Summary

Meiosis is a specialized cell division that produces haploid gametes. Homologous recombination plays a pivotal role in the segregation of homologous chromosomes during meiosis I by creating physical linkages between the chromosomes. Drastic reorganization of chromosomes in the nucleus is a prominent feature of meiotic prophase I, during which telomeres get associated with the nuclear envelope and move within the envelope, culminating in the formation of telomere clusters, often referred to as “chromosome bouquets.” The roles that telomere movement and clustering play in meiotic prophase I are largely unknown. In the budding yeast *Saccharomyces cerevisiae*, tethering of telomeres to the nuclear envelope is mediated by a meiosis-specific telomere-binding protein, Ndj1. We studied the functions of a meiosis-specific gene, *CSM4*, in telomere clustering and during meiotic recombination. *CSM4* is necessary for the clustering of Ndj1-associated telomeres. Interestingly, *csm4* mutants show pleiotropic defects during meiotic recombination. It is likely that the chromosome movement promotes various biochemical reactions during meiotic recombination.

## Introduction

Meiotic recombination promotes the faithful segregation of homologous chromosomes at meiosis I (MI) by creating physical linkages between the homologs [1],[2]. Recombination produces two types of products: crossovers (COs) and non-crossovers (NCOs). Only COs mature into exchanges between chromosome axes called chiasmata, which together with arm cohesion ensure homolog separation.

Recombination during meiosis is initiated by the formation of double-strand breaks (DSBs) at recombination hotspots [3]. A protein complex containing the Spo11 core catalytic subunit is involved in DSB formation. Resection of DSB ends results in the formation of single-stranded DNA (ssDNA), which is then used in the search for homologous DNA sequences. The homology search is catalyzed by two RecA homologs, Rad51 and Dmc1 with their accessory factors [4]–[7]. This homology search results in the invasion of ssDNA into duplex DNA, and the formation of a single-end invasion intermediates [SEIs; 8]. SEIs undergo second-end capture of the DSB to form a second prominent joint molecule, called the double-Holliday junction (dHJ), which is primarily resolved to form COs [9]. The intermediate required to form NCOs has yet to be identified. Importantly, the homology search resulting in SEI formation appears to be biochemically and temporally distinct from the second-end capture steps [8],[10].

CO formation is regulated by the action of a group of proteins called ZMM or SIC (synaptic initiation complex; hereafter called ZMM for simplicity). Members of the ZMM group include Zip1, Zip2, Zip3, Msh4, Msh5, Mer3, Spo16, and Spo22/Zip4 [11]–[16]. Mer3 and Msh4–Msh5 possess helicase and structure-specific DNA-binding activities, respectively [17],[18]. Zip3, together with the Zip2–Spo16–Spo22 adaptor complex, is thought to catalyze the post-translational modification of target protein(s), e.g., sumoylation or ubiquitylation [15],[19]. Zip1 is a component of the synaptonemal complex [20]. The ZMM proteins ensure the formation of wild-type CO levels [12],[16]. In addition to the ZMM-dependent CO pathway, budding yeast has two additional pathways for recombination: a minor CO pathway and a NCO pathway, both dependent on the junction resolvase Mus81–Mms4 [21],[22].

One of the most notable features in meiosis is chromosome dynamics and morphogenesis. In most organisms, synapsis of homologous chromosomes is facilitated by the recombination. Synapsis culminates in the formation of SC, a tripartite structure seen in pachytene [23],[24]. In leptotene when DSBs are formed, sister chromatids form chromatin loops along a shared axis (the axial element). Leptotene is followed by zygotene, in which short patches of SC form between homologous axial elements. Elongation of SC occurs along entire chromosomes, resulting in the formation of full-length SC in pachytene. SCs are then disassembled in the diplotene. Importantly, SC formation is tightly coupled with CO formation. Formation of SEIs and dHJs occurs at the leptotene-zygotene and zygotene-pachytene transitions, respectively [8],[12]. Resolution of dHJs occurs during late pachytene.

In the vegetative growth phase of *S. cerevisiae,* centromeres are present near the Spindle Pole Body (SPB), a fungal equivalent of the centrosome in other eukaryotes. In *S. cerevisiae*, the SPB is embedded in the nuclear envelope (NE), and telomeres are clustered and often associated with the NE in a dispersed distribution (Klein et al. 1992). This configuration of chromosomes in vegetative cells is referred to as the “Rabl” orientation. In meiotic prophase, cells undergo a drastic change in their chromosome configuration. Centromeres detach from the SPB, while telomeres cluster in one area of the nuclear membrane near the SPB. This chromosomal bouquet configuration is prominently seen only during zygotene. The bouquet is a conserved feature in the meiotic prophase of most eukaryotes, but its function remains unknown [23]. In *S. cerevisae*, a meiosis-specific telomere-binding protein, Ndj1 [25],[26], is involved in tethering telomeres to the nuclear membrane and promoting bouquet formation [27]. *ndj1* mutation reduces spore viability and confers some defects in recombination [25],[26],[28]. In *S. pombe*, the Bqt1–Bqt2 complex promotes bouquet formation through interactions with a telomere-binding protein, Taz1 [29]. The bouquet is thought to facilitate pairing of homologous chromosomes by restricting the homology search to a smaller area.

In this study, we found that a meiosis-specific protein, Csm4 [30], promotes efficient transition from SEIs to dHJs as well as resolution of dHJs in the CO-specific recombination pathway. These results suggest that Csm4 regulates various steps during meiotic recombination. Recombination-related phenotypes in *csm4* mutants are very similar to those seen in *ndj1* mutants [28]. Csm4 forms a complex with Ndj1 *in vivo*. We also found that similar to *ndj1* mutants, *csm4* mutants are deficient in bouquet formation, but unlike *ndj1* mutants, they are proficient in tethering telomeres to the NE. These results suggest that chromosome architecture and/or dynamics, which are mediated by the tethering telomeres to the NE, control various biochemical steps during meiotic recombination. The accompanying paper by Wanat et al. (2008) shows similar and complementary results [31].

## Results

### Csm4 Promotes Meiotic Recombination

**Figure 1:**
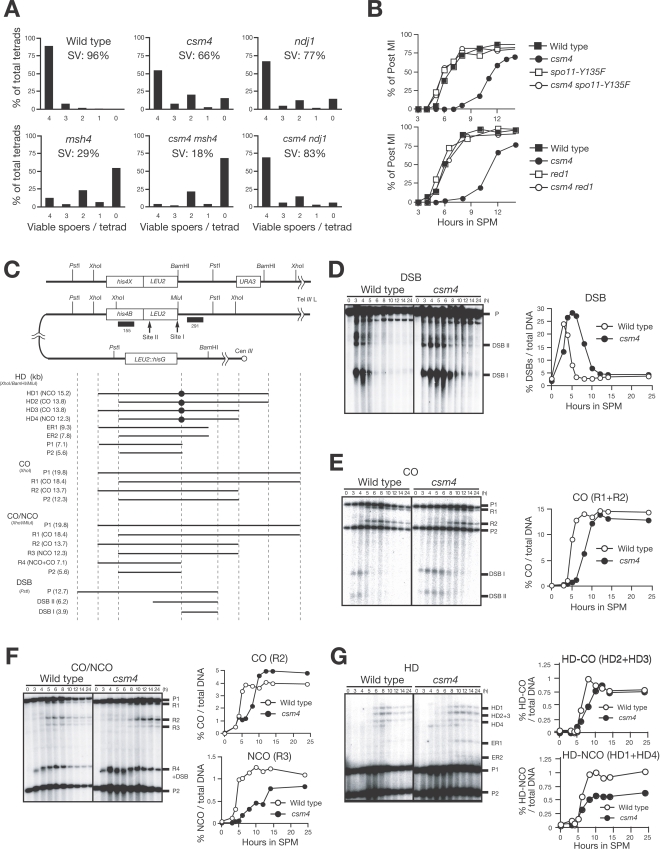
Csm4 promotes meiotic recombination. (A) Spore viability (SV). The indicated strains were sporulated at 30°C and more than 100 tetrads were dissected per strain. The distribution of 0, 1, 2, 3, and 4 viable spores per tetrad are shown for each strain. (B) Meiotic cell cycle progression. Entry into meiosis I and II in the wild type, *csm4* with or without *spo11-Y135F* (upper panel) and *red1* (bottom panel) mutations were analyzed by DAPI staining. Graphs show the percent of cells that completed MI at the indicated times. (C) A schematic diagram of the *HIS4-LEU2* recombination hotspot. Restriction sites for *Pst*I, *Xho*I, *Bam*HI, and *Mlu*I are shown. Diagnostic fragments for analysis on double-strand break (DSB), crossover (CO), non-crossover/crossover (CO/NCO) and heteroduplex (HD) in CO and NCO are shown at the bottom. The size of each fragment (kilo-bases) is presented within parentheses. (D-G) DSBs (D), CO (E), CO/NCO (F), and heteroduplex in COs and NCOs (G) at the *HIS4-LEU2* locus in the wild type and *csm4* cells were analyzed by Southern blotting and quantified (graphs on right). Genomic DNA was digested as follows; DSBs, *Pst*I; CO, *Xho*I; CO/NCO, *Xho*I and *Mlu*I; heteroduplexes, *Xho*I, *Bam*HI and *Mlu*I. ER (in G) is a product of intra-chromosomal ectopic recombination. Wild type, open circles; *csm4* mutant, closed circles.

Previous analysis showed that *csm4* mutants are defective in the segregation of chromosomes during meiosis [30]. However, little is known about the functions of Csm4 in meiosis. We re-analyzed the meiotic phenotypes of *csm4* mutants in an SK1 background. Consistent with a previous study [30], the *csm4* mutation reduces spore viability to 66%, as compared to 96% in the wild type. Interestingly, 4-, 2-, and 0-viable spore tetrads exceed 3- and 1-viable spore tetrads, suggesting non-disjunction of homologs at MI (Figure 1A) Similar results have been described by Wanat et al. in the accompanying paper [31]. Furthermore, *csm4* mutation delays its entry into MI by 5 h (Figure 1B). This delay is suppressed by introducing a mutant allele of *SPO11*, *spo11-Y135F*, which abolishes the catalytic function [3],[32]. Similar results have been described by Wanat et al. [31], suggesting that the delay seen in the *csm4* mutant is due to a defect in meiotic recombination. The delay is also suppressed by the introduction of a mutation of the *RED1* gene (Figure 1B), which encodes a component of the axial element of the SC [33], is necessary for DSB formation [34], and acts as a barrier to inter-sister recombination [35],[36].

We then analyzed the turnover of meiotic DSBs at the *HIS4-LEU2* recombination hotspot [37] in the *csm4* mutant (Figure 1C). In the wild type, DSBs appear at 3 h after incubation in the sporulation medium (SPM) and then disappear at around 6 h (Figure 1D). The *csm4* mutant accumulates DSBs up to a slightly higher level compared to the wild type. Formation of DSBs in the mutant is slightly delayed, and disappearance of the DSBs is delayed by 4 h. At 8 h, DSBs are still detected in the mutant. These results indicate that *CSM4* is required for the efficient conversion of DSBs into later-stage recombination intermediates.

Next, we examined the formation of crossovers (COs) in *csm4*. Consistent with delayed DSB repair, CO formation in *csm4* is delayed by approximately 4 h compared to the wild type (Figure 1E). Similar results have been described by Wanat et al. in the accompanying paper [31]. However, the final level of COs at the *HIS4-LEU2* locus is similar to the wild type (92% of the wild-type level).

In addition to COs, meiotic recombination produces non-crossovers (NCOs). CO and NCO recombinants can be distinguished using restriction site polymorphisms around DSB site I in the *HIS4-LEU2* locus [38]. As seen for COs, NCOs in the *csm4* mutant are formed 5 h later than in the wild type (Figure 1F). In this assay, the final level of COs in the mutant is slightly higher (1.2-fold) than the wild type. Wanat et al. show a slight reduction of NCOs using the same assay [31]. The level of NCOs in *csm4* is reduced to 75% of the wild type. This suggests that Csm4 is required for timely and efficient formation of both types of recombinants. This was confirmed using a heteroduplex assay that detects CO and NCO at the same locus (Figure 1G). The final level of NCOs containing heteroduplex DNA at the *Mlu*I/*Bam*HI site in the mutant is reduced to 50% that of the wild-type level, while the level of COs containing heteroduplex DNA is unaffected by *csm4* mutation. Interestingly, the *csm4* mutant increases ectopic recombination between *HIS4-LEU2* and *leu2::hisG* on chromosome *III* (Figure 1G; [39]).

### Relationship of Csm4 with Msh4 and Mms4 during Meiotic Recombination

**Figure 2:**
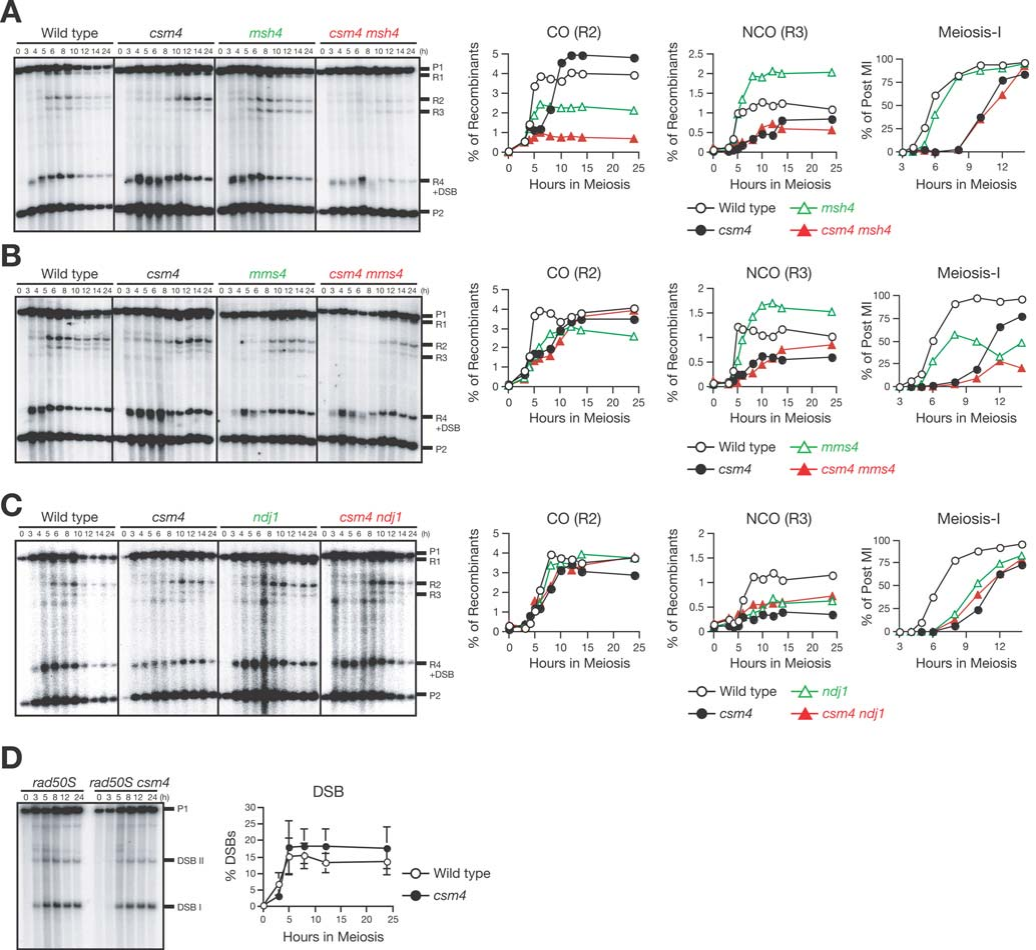
Relationship of *csm4* with *msh4*, *mms4*, and *ndj1* in meiotic recombination. (A-C) CO/NCO analysis of the wild type, *csm4* mutant with or without *msh4* (A) *mms4* (B), or *ndj1* (C) null mutant allele. Southern blots were prepared as shown in Figure 1 and the quantification of COs and NCOs as well as progression through meiosis I (MI) in each strain is shown in the graphs (right). Progression through MI was analyzed by DAPI staining. Wild type (A, B, C), open circles; *csm4* (A, B, C), closed circles; *msh4* (A), *mms4* (B), *ndj1* (C), open green triangles; *csm4 msh4* (A), *csm4 mms4* (B), *csm4 ndj1* (C), closed red triangles. (D) DSBs at the *HIS4-LEU2* locus in *rad50S* and *csm4 rad50S* cells were analyzed by Southern blotting (left) and quantified (right) as described in the Materials and Methods. Error bars (+/−SD) were obtained from three independent analyses.

Meiotic recombination has been grouped into two CO pathways and a single NCO pathway [21]. One major pathway for COs depends on ZMM proteins [12] and the other depends on the junction-specific resolvase, Mus81–Mms4 [22]. To examine a possible role for Csm4 in these pathways, we constructed a *csm4* mutant with a mutation in *MSH4*, which encodes a meiosis-specific MutS homolog that acts in the ZMM pathway [40]. *csm4* and *msh4* single mutants display reduced spore viability (66 and 29%, respectively; Figure 1A). The *csm4 msh4* double mutant shows more severe defects in spore viability (18%) than either single mutant. In the CO/NCO assay, *msh4* affects formation of both COs and NCOs (Figure 2A). As reported previously [12], at 30°C, *msh4* mutation decreases the final amount of COs to 50% that of the wild type, but increases the level of NCOs to 1.7-fold of the wild type. The *csm4 msh4* double mutant shows more severe defects in CO formation; the final level of COs in the double mutant is significantly reduced compared to *csm4* and *msh4* single mutants. However, the amount of NCOs in the *csm4 msh4* double mutant is only slightly reduced compared to the *csm4* single mutant. This suggests that Csm4 functions in meiotic recombination independently of Msh4 and that Csm4 promotes CO formation in the absence of Msh4. Furthermore, Msh4 is not necessary for residual NCO formation in the absence of Csm4.

Next, we constructed the *csm4 mms4* double mutant. Unlike either single mutant, the *csm4 mms4* double mutant cannot form spores. In the CO/NCO assay (Figure 2B), the *mms4* single mutant exhibits a delay in formation of COs and reduces CO levels to 73% of the wild type [41]. Interestingly, NCOs in the *mms4* mutant appear at the same time as in the wild type and at levels that are 1.5-fold higher than the wild type. The *csm4 mms4* double mutant shows an effect on NCO formation similar to the *csm4* single mutant. Similar to *csm4*, CO formation in the double mutant is delayed, but reaches an almost wild-type level. These observations suggest that Csm4 works upstream of Mms4 in meiotic CO and NCO recombination pathways.

We also examined the amount of DSBs formed in the *csm4* mutant in the *rad50S* background, which blocks processing of DSB ends [42]. The *csm4 rad50S* double mutant accumulates DSBs like the *rad50S* mutant (Figure 2E). Similar results have been described by Wanat et al. in the accompanying paper [31]. DSB levels in the double mutant were slightly higher than those seen in *rad50S*.

### Csm4 Is Required for Timely Formation of and Exit from Double-Holliday Junctions

**Figure 3:**
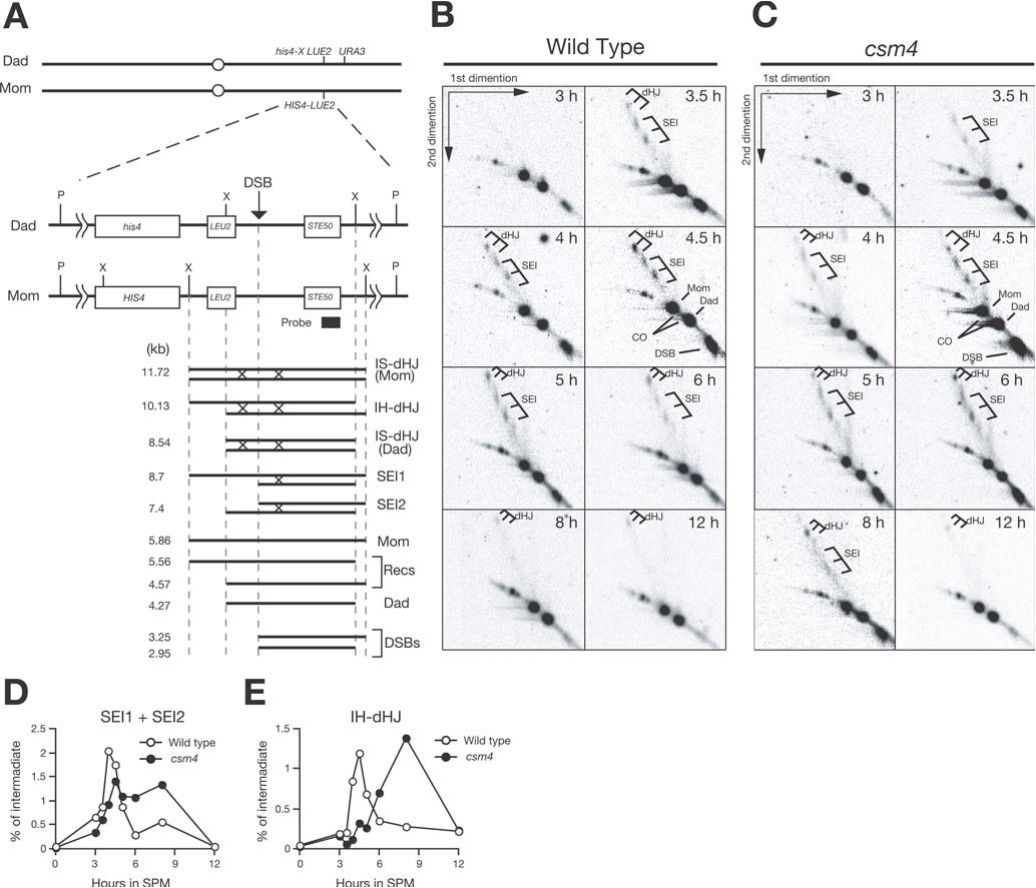
2D analysis of recombination intermediates in the *csm4* mutant. (A) Schematic drawing of the *HIS4-LEU2* locus for the SEI and dHJ assay. (B, C) Southern blots of 2D gel analysis of the wild type (B) and the *csm4* mutant (C). Genomic DNA samples taken at various times were psoralen-crosslinked, digested with *Xho*I, and analyzed in 2D neutral/neutral gels. (D, E) Quantification of SEI and dHJ. The amount of SEIs (D) and interhomolog dHJs (E) were quantified at each time point and plotted. Wild type, open circles; *csm4*, closed circles.

As shown above, Csm4 is necessary for timely CO formation recombination pathway, which mainly depends on ZMM proteins such as Msh4. In the ZMM-dependent CO pathway, single-end invasions (SEIs) and double-Holliday junctions (dHJs) have been identified as major recombination intermediates [8],[9]. We analyzed the effect of *csm4* mutation on the formation of these intermediates, which can be detected at *HIS4-LEU2* (Figure 3A) in 2D gel electrophoresis after cross-linking DNA samples with psoralen [8],[9]. In the wild type, SEIs begin to appear at 3 h, peak at 4.5 h, and disappear at around 6 h (Figure 3B and 3D). In contrast, the *csm4* mutant shows a slight delay in the onset of SEI formation, and SEIs persist at later times during meiosis (Figure 3C and 3D). At 8 h, a significant level of SEIs could be detected in the *csm4* strains. Although delayed, SEIs are turned over in the mutant at around 12 h. dHJs in the wild-type cells start to appear at 4.5 h, peak at 5 h, and then disappear (Figure 3B and 3E). In *csm4*, formation of dHJs is delayed by 3.5 h compared to the wild type (Figure 3C and 3E). The maximum level of dHJs in the mutant at 8 h is slightly higher than in the wild type. Furthermore, the resolution of dHJs is clearly delayed in the mutant. These data suggest that *csm4* mutation affects various steps of CO formation, likely during the SEI–dHJ transition and dHJ resolution. Similar results but with more quantitative analysis of recombination intermediates have been described in the accompanying paper by Wanat et al. [31].

### Csm4 Is Necessary for Timely Disassembly of RecA Homolog Foci and Efficient Chromosome Synapsis

**Figure 4:**
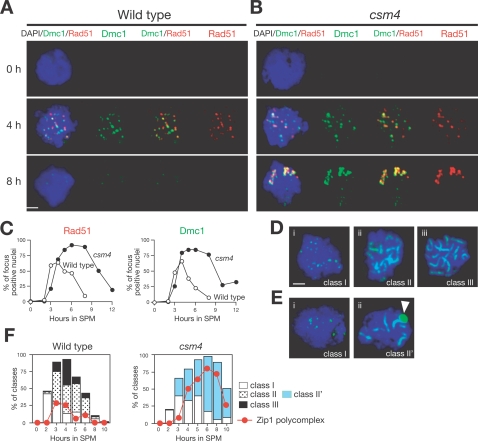
The *csm4* mutant is defective in disassembly of RecA homolog foci and SC formation. (A–C) Rad51-Dmc1 focus formation in the *csm4* mutant. Nuclear spreads of the wild type (A) and *csm4* (B) were stained with anti-Rad51 (red; left graph) and anti-Dmc1 (green; right graph) as well as DAPI for DNA (blue). The percent of cells positive for Rad51 or Dmc1 foci (more than 5 foci per nucleus) were counted at each time point (C). At least 100 nuclei were counted at each time point. Wild type, open circles; *csm4* mutant, closed circles. Bars = 2 µm. (D, E) Chromosome synapsis in *csm4* mutants. Nuclear spreads were stained with anti-Zip1 (green) and DAPI (blue), categorized, and plotted as described previously [33]. SCs of wild-type cells shown in leptotene (D-i), zygotene (D-ii), and pachytene (D-iii). SCs of *csm4* mutants shown in leptotene (E-i) and zygotene-like stages (E-ii) contain the polycomplex (PC) as shown by an arrow. Bars = 2 µm. (F) Plots showing each class of SC (Wild type, left; *csm4* mutant, right) at indicated times in meiosis. Class I (open bars), Zip1 dots; Class II (dotted bars), partial Zip1 linear; Class III (closed bars), linear Zip1 staining; Class II', partial Zip1 linear with PC (blue bars). The formation of Zip1 PC is shown for each strain (red closed circles).

We analyzed the localization of RecA homologs on meiotic chromosome spreads by immunostaining. Eukaryotic RecA homologs Rad51 and meiosis-specific Dmc1 both act in the homology search/strand exchange process that results in SEI and dHJ formation [6],[43],[44]. In the wild type, Rad51 as well as Dmc1 shows punctate staining, or foci [44],[45]. Rad51 foci begin to appear at 3 h, peak at 4 h, and then disappear at later times (Figure 4A). The kinetics of Rad51 focus formation is very similar to that of DSBs. In the *csm4* mutant, the formation of Rad51 foci is slightly delayed compared to the wild type (Figure 4B and 4C), consistent with a delay in DSB formation in the mutant. Disassembly of Rad51 foci is clearly delayed in the *csm4* mutant, indicating inefficient repair of DSBs. The average number of Rad51/Dmc1 foci in the *csm4* mutant at 4 h is 42.8 for Rad51 and 40.5 for Dmc1 (per total nucleus), which is higher than that seen in the wild type (22.6 and 24.8 for Rad51 and Dmc1, respectively). At later time points, much brighter and larger Rad51 foci, possibly representing aggregates, are observed in the mutant (Figure 4B). These aggregates appear to be specific to *csm4*, since other mutants, which also accumulate Rad51/Dmc1 foci at later times (e.g., *tid1*, *mnd1*, and *hop2*), do not accumulate these structures [45]–[47]. Dmc1 in *csm4* shows a staining pattern similar to that seen for Rad51 (Figure 4B and 4C). These data suggest that *CSM4* is necessary for a step after loading of Rad51 and Dmc1, e.g., during the homology search.

To examine the effect of *csm4* on chromosomal synapsis, i.e., formation of the synaptonemal complex (SC) during meiotic prophase, we stained chromosome spreads with an antibody against the Zip1 protein, which is a component of the central element of the SC [20]. In leptotene, Zip1 shows dotty-staining in the wild type (2–3 h; class I; Figure 4Di). In zygotene (3–5 h), short lines of Zip1 (class II; Figure 4Dii) are observed in addition to the Zip1 foci. At pachytene (5–7 h), Zip1 elongates along entire chromosomes (class III; Figure 4Diii), indicating full chromosome synapsis. The *csm4* mutant shows a deficiency in SC formation. Similar to the wild type, Zip1 foci form in the mutant (Figure 4Ei). Zip1 starts to elongate, but full chromosome synapsis is rarely seen in the mutant (class II'; Figure 4Eii). As a result, the *csm4* mutant accumulates zygotene-like nuclei (Figure 4F). Consistent with a synapsis defect, most zygotene-like *csm4* nuclei contain an aggregate of Zip1 called polycomplex. Although pachytene-like nuclei are rare in the mutant, Zip1 dismantles when further incubated with SPM (Figure 4F). These results indicate that *CSM4* is required for efficient SC formation, particularly SC elongation. Similar results have been described by Wanat et al. using Zip1–Green fluorescent protein (GFP) fusion protein [31].

### Csm4 Interacts with Meiosis-Specific Telomere-Binding Protein, Ndj1

**Figure 5:**
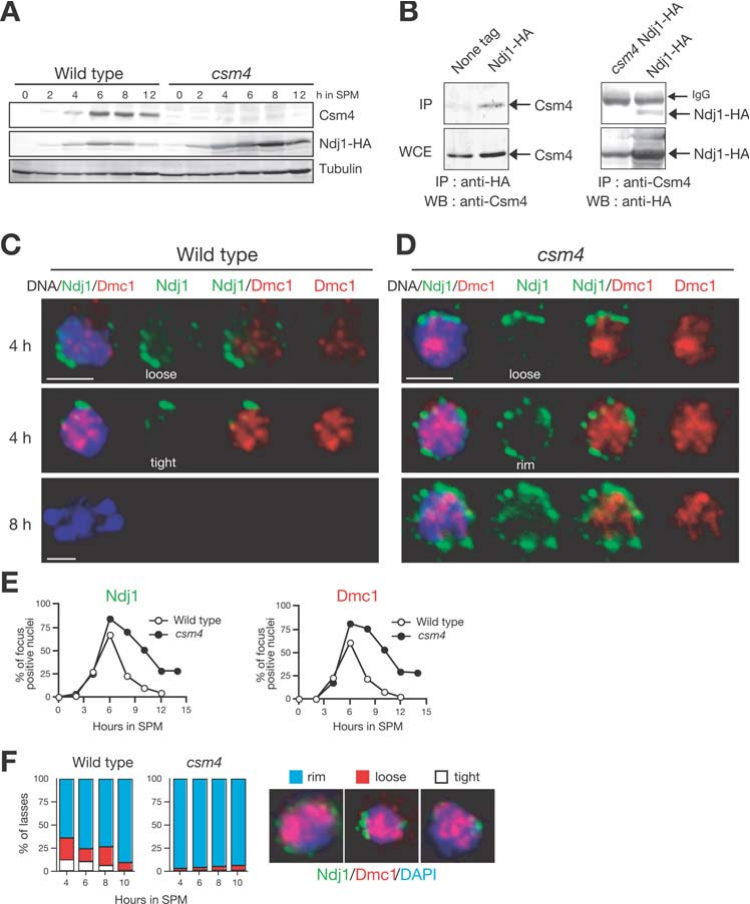
Csm4 promotes the clustering of Ndj1 at the nuclear periphery. (A) Expression of Csm4 protein. Lysates obtained from the wild type and *csm4* mutant strains bearing *NDJ1-HA* induced for meiosis were analyzed by Western blotting using anti-Csm4 (upper), anti-HA (middle), or anti-tubulin (lower) antibodies. (B) Co-immunoprecipitation of Csm4 and Ndj1. Cell lysates from strains containing or lacking *NDJ1-HA* were immunoprecipitated with anti-HA (left) and anti-Csm4 (right) antibodies, and probed with anti-Csm4 (left upper panel) and anti-HA (right upper panel), respectively. Whole cell extracts (WCE; bottom panels) were also analyzed by Western blotting. (C, D) Localization of Ndj1 protein in intact meiotic cells. Wild type (C) and *csm4* mutant strains (D) containing *NDJ1-HA* were induced for meiosis. Cell aliquots were collected at indicated times, fixed, stained with anti-HA and anti-Dmc1 antibodies, and examined using a fluorescence microscope. Ndj1-HA (green), Dmc1 (red), and DAPI (blue). Bars = 2 µm. (E) Kinetics of Ndj1 and Dmc1 foci. Nuclei positive for Ndj1 (left) and Dmc1 (right) localization in whole cells (C, D) were counted and plotted. Wild type, closed circles; *csm4* mutant, open circles. (F) Classes of Ndj1–telomere clustering. Cells with tight bouquet, loose bouquet, or peripheral staining of Ndj1-HA (green) are classified at each time after induction of meiosis. The percent of each class (per Ndj1-positive cells) is shown for the wild type (left) and the *csm4* mutant (right).

Expression of *CSM4* mRNA is specific to meiosis [30]. Western blotting analysis using an antibody against Csm4 reveals that this protein is present in lysates from meiotic cells, but not from mitotic cells (Figure 5A). Our initial immunostaining analysis of both whole cells and chromosome spreads failed to localize the protein either in nuclei or on chromosomes (HK, unpublished results). However, when expressed in vegetative cells as a GFP fusion protein, Csm4 localizes to nuclear membranes and the endoplasmic reticulum [48].

We noticed that the *csm4* and *ndj1* mutants share similar recombination defects [28]. In particular, similar to *csm4*, the *ndj1* mutant specifically decreases NCO formation in physical assays. When a *csm4 ndj1* double mutant was constructed and analyzed for CO/NCO formation, the double mutant exhibited a phenotype similar to *csm4* and *ndj1* single mutants (Figure 2C). Although *CSM4* and *NDJ1* appear to function in the same recombination pathway, there are several phenotypic differences between two single mutants. In general, *csm4* shows more severe defects than *ndj1* and *csm4 ndj1* double mutants show defects that are more similar to *csm4*. The spore viability of *csm4* is lower than that of *ndj1* (Figure 1A; 66% versus 77%), and the *csm4* mutant enters into MI 2 h later than the *ndj1* mutant (Figure 2C).

Ndj1 is a meiosis-specific protein that binds to telomeres [25],[26] and is required to form the bouquet, where telomeres cluster near the SPB [27]. The similarity between *csm4* and *ndj1* phenotypes prompted us to examine the interaction of Csm4 with Ndj1. We used a strain in which Ndj1 protein is tagged with the HA epitope at its C-terminus. This strain exhibits wild-type spore viability. Immunoprecipitation (IP) using anti-HA antibody reveals the presence of Csm4 in precipitates of meiotic cell lysates from *NDJ1-HA* diploid, but not in those from the untagged strain (Figure 5B). Reciprocal IP using anti-Csm4 also detects Ndj1-HA in these precipitates (Figure 5B). These results demonstrate a physical association interaction of Csm4 with Ndj1 in meiotic cells. Since the *csm4* mutant expresses Ndj1 (Figure 5A), the defect conferred by *csm4* is not due to the inability of *csm4* cells to express Ndj1.

### *CSM4* Promotes Proper Clustering of Ndj1

Next, we studied the localization of Ndj1-HA protein to the NE in *csm4* mutants. Whole cells were fixed with formaldehyde and then stained with anti-HA antibody followed by fluorescent-conjugated antibody. The cells were then observed under an epifluorescence microscope. We also analyzed the localization of Dmc1 in intact cells as a marker for meiotic cells. As reported previously [27], in wild-type cells, Ndj1 shows several foci or patches near the nuclear periphery in the meiotic prophase (Figure 5C). The kinetics of accumulation and disappearance of Ndj1- and Dmc1-positive cells were very similar (Figure 5E). We sorted the staining patterns into three classes: rim, loose bouquet, and tight bouquet (Figure 5F). Loose and tight bouquets are only seen in the meiotic prophase (Figure 5F). Furthermore, a significant fraction of wild-type cells at 4 h shows clustering of Ndj1 foci (loose and tight bouquets) in one area of the NEs (Figure 5F). On the other hand, *csm4* cells do not show clustering of Ndj1, but rather exhibit dispersed staining of Ndj1 patches at the nuclear periphery (Figure 5D and 5F). In *csm4*, Ndj1 patches persist in the periphery longer than in the wild type (Figure 5D and 5E). Importantly, Ndj1 in the *csm4* mutant is still associated with the NE. These data indicate that Csm4 is required for efficient clustering of Ndj1 on the NE, but not for tethering, suggesting a role of Csm4 in Ndj1-mediated telomere clustering.

### *CSM4* Promotes Bouquet Formation

**Figure 6:**
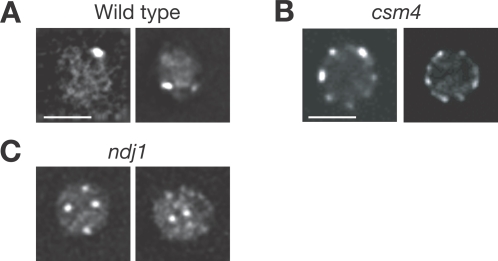
Csm4 promotes bouquet formation. Localization of Rap1–GFP in intact cells. Wild type (A), the *csm4* mutant (B) and *ndj1* mutant cells (C) with Rap1–GFP were directly examined as described in Materials and Methods. Rap1–GFP is shown in white. Deconvoluted images of one focal plane are shown. Bars = 2 µm.

Ndj1 promotes telomere clustering during meiotic prophase [27]. We examined bouquet formation by analyzing Rap1–GFP localization [49],[50]. Rap1 is concentrated at telomeres and is used as a marker for telomere localization [51]. As shown previously, Rap1–GFP is localized at the nuclear periphery as several foci in mitosis [49],[52]. Nuclei were visualized by deconvoluting Z-series images; one focal plane is shown in Figure 6. When diploid cells enter the meiotic prophase, after 3–5 h incubation with SPM, a small fraction of diploid cells in meiotic prophase show a polarized distribution of Rap1–GFP at the cell periphery. In *S. cerevisiae*, the bouquet appears unstable and is possibly dynamic during the meiotic prophase [50],[52]. In the wild type, clustering of Rap1 foci is prominently seen at 4 h (Figure 6A); however, due to the very transient nature of the clustering, only 15–25% cells show the clustering. On the other hand, the *csm4* mutant shows a disperse distribution of Rap1 on NEs after 4 and 5 h incubation with SPM (Figure 6B). Very few cells show the clustering of Rap1–GFP in the mutant between 4 and 6 h (see Figure 5F). This indicates that Csm4 is necessary for clustering of telomeres but not for tethering telomeres to the NE. Furthermore, some Rap1–GFP foci in the *ndj1* mutant are not localized at the nuclear periphery but are seen within the nucleus [Figure 6C; 27]. Similar results have been described by Wanat et al. [31]. We also noticed that most *csm4* nuclei were round, while the wild type as well as the *ndj1* mutant nuclei were irregularly shaped, suggesting a defect in nuclear deformation in the *csm4* mutant.

### *CSM4* Is Not Required for Relocalization of Mps3

**Figure 7:**
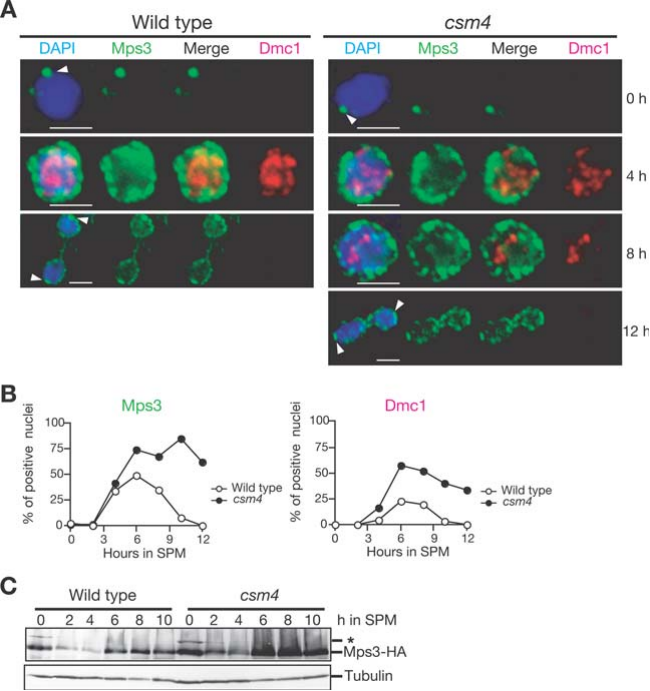
Mps3 relocalization is independent of Csm4. (A) Localization of Mps3-HA protein in intact meiotic cells. Wild type (left) and *csm4* mutant (right) bearing *MPS3-HA* were induced for meiosis, collected at the indicated times, fixed, stained with anti-HA and anti-Dmc1 antibodies, and then examined using a fluorescence microscope. Mps3-HA (green), Dmc1 (red), and DAPI (blue). Arrowheads show the possible location of the SPB. Bars = 2 µm. (B) Appearance and disappearance of Mps3- and Dmc1-positive cells. Nuclei positive for Mps3 (left) and Dmc1 (right) localization in whole-cell staining were counted and plotted. Wild type, open circles; *csm4* mutant, closed circles. (C) Expression of Csm4 protein. Cell lysates prepared from the wild type and *csm4* mutants bearing the *MPS3-HA* at indicated times in meiosis were analyzed by Western blotting using anti-HA (upper panel) or anti-tubulin (lower panel) antibodies. An asterisk indicates a non-specific band.

It was recently reported that a component of the SPB, Mps3, is necessary for telomere clustering during meiosis [53] and anchoring telomeres [54]. Mps3, which contains Sad1-Unc-84 (SUN) and trans-membrane domains, changes its localization from the SPB to the NE during meiosis [53],[54]. We examined the effect of *csm4* mutation on Mps3 relocalization. Whole cells containing *MPS3* tagged with HA were fixed with formaldehyde and stained with antibodies against the HA tag and Dmc1 protein. As reported previously [54],[55], at 0 h, Mps3 is seen as a single spot at the nuclear periphery (Figure 7A), which is consistent with its localization near the SPB. During the meiotic prophase (at 4 h in SPM), in Dmc1-positive nuclei, Mps3 relocalizes throughout the NE and occasionally exhibits patchy staining (Figure 7A). This NE localization of Mps3 is still observed after the MI division. In the *csm4* mutant, Mps3 shows a distribution in the NE similar to the wild type, but remains longer than in the wild type (Figure 7A and 7B). This is consistent with a prolonged meiotic prophase in *csm4*. Therefore, the effect of the *csm4* mutation on telomere clustering appears to be independent of Mps3 relocalization. In addition, *csm4* does not affect Mps3 protein levels (Figure 7C).

## Discussion

### Csm4 Functions with Ndj1

Previously, the *csm4* mutant was isolated on the basis of its defect in chromosome segregation during meiosis [30]. In this paper, we show that Csm4 functions with the meiosis-specific telomere-binding protein Ndj1. Recombination defects conferred by a *csm4* mutation are very similar to those caused by a mutation in *NDJ1* [28]. Indeed, the *csm4 ndj1* double mutant phenotype is similar to that seen for the single mutants. In addition, co-IP shows that Csm4 is physically associated with Ndj1 *in vivo*. Furthermore, Csm4 is required for efficient clustering of Ndj1 at the nuclear periphery. These results indicate that Csm4 and Ndj1 function in the same structural pathway.

The *csm4* mutant, however, shows more severe defects in meiosis than the *ndj1* mutant. Spore viability is lower in *csm4* compared to *ndj1*. The *csm4* mutation delays its entry into MI to a greater extent than *ndj1*. These observations suggest that Csm4 has additional functions in meiosis or that *ndj1* is not null for related functions.

### *CSM4* Is Necessary for Various Steps of Meiotic Recombination Pathways

Csm4 is necessary for normal functioning of all three recombination pathways of meiosis: ZMM-dependent and -independent (*MMS4*-dependent) CO and NCO formation. Although the final level of COs in the *csm4* single mutant is similar to that in the wild type, *csm4* reduces the level of NCOs compared to the wild type, indicating the involvement of Csm4 in NCO formation during meiosis. Csm4 is a meiosis-specific protein; this suggests that NCO formation is under the control of a meiotic program and thus is likely to be mechanistically distinct from NCO formation during mitosis. When *csm4* mutation is combined with a mutation in *MSH4*, the double mutant is almost completely deficient in CO formation. Therefore, Csm4 functions in CO formation independently of Msh4.

CO formation in meiosis, about half of which depends on *ZMM* genes, is severely delayed in *csm4*, indicating that Csm4 is also required for efficient formation of COs in the major *ZMM*-dependent meiotic recombination pathway. Two intermediates, SEIs and dHJs, have been identified in the *ZMM*-dependent CO pathway [8],^9^. The most severe effect of *csm4* mutation is seen in SEI–dHJ transition and dHJ resolution, two distinct biochemical steps in the *ZMM* pathway. It is likely that SEI–dHJ transition is accompanied by the capture of SEI by the second end of the DSB [8],[56]. Therefore, Csm4 seems to promote the second-end capture during strand exchange. Generally, this capture is considered a simple annealing reaction between ssDNA of the second end and a displaced ssDNA in SEI [57]. However, our results strongly suggest that the second-end capture is not a simple biochemical reaction as believed previously; rather, it is a critical regulatory step in the CO pathway. Although the exact molecular nature of SEIs in the *csm4* mutant is not known, they are likely to contain D-loop structures that can be converted into COs or NCOs (Hunter et al. 2002). Thus, the transition of SEIs to dHJs could be regarded as an irreversible commitment step towards CO formation. The transition can be independently governed by both ZMM- and Csm4-dependent functions. Furthermore, disassembly of the two RecA homologs Rad51 and Dmc1 is delayed in the *csm4* mutant. This strongly suggests that disassembly of the RecA homologs occurs during the SEI–dHJ transition, and thus is somehow coupled with the second-end capture.

### Csm4 Affects Meiotic Recombination through Telomeres

How does Csm4 control the various steps during meiotic recombination? One notion is that Csm4 functions as an enzyme directly involved in recombination. However, it is very difficult to assign a biochemical activity to Csm4 (∼23 kD) with no apparent structural domains, since it is likely to be involved in various steps in the aforementioned three recombination pathways. One possibility is that Csm4 acts as a component of the meiotic chromosomes. Red1, a chromosome axis protein, is involved in various recombination steps [43],[58]. However, our initial attempt to localize the protein on DNA by chromatin IP failed to detect the binding of Csm4 to a recombination hotspot (unpublished results). Our initial attempt to localize Csm4 was also unsuccessful because both N- and C-terminal tagged genes are non-functional and our anti-Csm4 did not work for immunostaining (HK, unpublished results). However, Csm4 is predominantly enriched in the NE when overexpressed as a GFP fusion protein in vegetative cells [48], consistent with the fact that Csm4 contains a putative transmembrane domain. Furthermore, a Csm4 partner, Ndj1, is enriched at telomeres, that are tethered to the NE [25],[26]. These observations strongly suggest that Csm4 is localized in the telomeres. Indeed, similar to Ndj1, Csm4 binds to telomeres on nuclear spreads [59]. Thus, the Csm4–Ndj1 complex is likely to affect recombination indirectly through its function at telomeres and/or the NEs. In addition to Csm4–Ndj1, the Mps3 protein containing Sad1-UNC84 domain is also involved in the process [53]. During vegetative growth, Mps3 is localized to the SPB and then relocated to the NEs in the meiotic prophase [53]. Mps3 forms a complex with Ndj1 and Csm4 [59]. An allele of *mps3* shows pairing defects in meiosis similar to those seen in *ndj1* and *csm4* mutants [59]. Given that Mps3 is an inner nuclear membrane protein, it is likely to tether Ndj1-bound telomeres to the NE.

### How Does Telomere Tethering to the NE Regulate Meiotic Recombination?

How do telomeres control recombination on the interstitial sites of chromosomes? The fact that *ndj1* and *csm4* mutants are defective in chromosomal bouquet formation [this study; 59],[60] suggests that a polarized configuration of chromosomes in zygotene might play a positive role in meiotic recombination. As proposed previously [23],[61], telomere clustering may restrict the arrangement of chromosomes in the nucleus, and in turn increases the probability that two allelic loci undergo colocalization. Although this could explain defects specific to zygotene, such as first end capture or SEI formation, those in second-end capture and dHJ resolution, occurring in the end of zygotene and pachytene, respectively, cannot be simply explained by telomere clustering during zygotene.

Rather, we propose that chromosome dynamics accompanied by telomere movement facilitates meiotic recombination. Tethering telomeres to nuclear membranes followed by movement along the envelope might change the chromatin structure, which might indirectly promote various biochemical steps during recombination. Dynamic movement of chromosomes in the meiotic prophase has been recently described; it depends on actin polymerization [59],[60],[62],[63]. Furthermore, the dynamic nature of telomeres on the NE is somehow dependent on Ndj1 and Csm4 [59],[60]. It is likely that the global changes in the chromosome structure and/or movement of chromosomes, promoted by the anchoring of telomeres to the NE, control the biochemistry of recombination of meiotic chromosomes.

### Multi-Step Assembly of Chromosomal Bouquet in Budding Yeast

Our analysis of *csm4* provides new insights into the mechanism of telomere clustering in budding yeast. Both *csm4* and *ndj1* mutants are deficient in telomere clustering, but the nature of deficiency in these mutants is qualitatively different. While *NDJ1* promotes tethering of telomeres to the NE, *CSM4* facilitates clustering of Ndj1-bound telomeres in one area of the envelope. Csm4 may promote bouquet formation by directly clustering the telomeres and/or by stabilizing them. Given that telomere movement on the envelope is a dynamic process [52],[62], Csm4 might be involved in the movement of telomeres on the NE. However, the *csm4* mutant exhibits some local movement of telomeres on the membrane, which is clearly different from the movement in the presence of an actin-inhibitor [60; HK and AS, unpublished results]. Thus, the movements of telomeres are either Csm4-dependent or Csm4-independent. Our results suggest that meiotic telomere clustering consists of different steps including telomere tethering, movement, and clustering. Consistent with this, nuclei in *csm4* mutants are relatively round compared to the irregular shapes of meiotic nuclei seen in the wild type (Figure 6). Nuclear deformation may be induced by external physical forces on the nuclei. Therefore, Csm4 might be involved in the transduction of forces on the NEs.

## Methods

### Strains and Plasmids

All strains described here are derivatives of SK1 diploids, NKY1551 (*MATα/MAT**a**, lys2/lys2, ura3/ura3, leu2::hisG/leu2::hisG, his4X-LEU2-URA3/his4B-LEU2, arg4-nsp/arg4-bgl*) and NKY3230 (*MATα/MAT**a**, lys2/lys2, ura3/ura3, leu2::hisG/leu2::hisG, his4X-LEU2-(N/Bam)-URA3/HIS4-LEU2-(N/Bam)* and its derivatives with *csm4::KamMX6* were used for the 2D analysis. Rap1-GFP was a kind gift from Dr. Y. Hiraoka. The genotypes of each strain used in this study are described in Table S1.

### Strain Construction

*csm4, ndj1, mms4,* and *msh4* null alleles were constructed by PCR-mediated gene disruption using either the *URA3* gene or the *KanMX6* [64]. *NDJ1-3HA* and *MPS3-3HA* were constructed by a PCR-based tagging methodology [65].

Primer details used for PCR amplification are available upon request.

### Anti-Serum Preparation and Antibodies

Anti-Csm4 antibody was raised against recombinant protein purified from *E. coli*. The open reading frame of Csm4 was PCR-amplified and inserted into pET15b plasmid (Novagen) in which the N-terminus of *CSM4* gene was tagged with hexahistidine. Csm4 protein with the histidine tag was affinity-purified in accordance with the manufacturer's protocol and used for immunization (MBL Co. Ltd). Primer details for PCR amplification are available upon request.

Anti-HA antibody (16B12; Babco), anti-tubulin, guinea pig anti-Rad51 [45], and rabbit anti-Dmc1 [5] were used for staining. Antiserum against Zip1 was raised using a recombinant GST-fusion protein purified from *E. coli* [16].

### Cytology

Immunostaining of chromosome spreads was performed as described previously [45],[66]. Whole cell immuno-staining was preformed as described previously [27] with a slight modification. Cells were fixed with formaldehyde. Stained samples were observed using an epi-fluorescent microscope (BX51; Olympus) with a 100x objective (NA 1.3). Images were captured by a CCD camera (Cool Snap; Roper), and processed using IP lab (Sillicon) and Photoshop (Adobe) software. For focus counting, more than 100 nuclei were counted at each time point.

Rap1-GFP was observed as described previously [52]. Images were captured by a computer-assisted fluorescence microscope system (Delta Vision; Applied Precision) with an oil-immersion objective lens (100x, NA 1.35). Image deconvolution was performed using an image workstation (SoftWorks; Applied Precision).

### Analyses of Meiotic Recombination

Time-course analyses of DNA events in meiosis and cell cycle progression were performed as described previously [8],[12],[58].

### Immuno-Precipitation Assay and Western Blotting

IP assay was performed as described previously [5].

### Reproducibility

Each result presented in the figures is representative of several experiments. The number of experiments performed is shown in Table S2.

## Supporting Information

Table S1 Strain list.
(0.04 MB DOC)

Table S2 The number of experiments.
(0.04 MB DOC)
