# Supplementary material for: Csm4-Dependent Telomere Movement on Nuclear Envelope Promotes Meiotic Recombination
Source: PLoS Genet. 2008 Sep 26;4(9):e1000196. doi: 10.1371/journal.pgen.1000196 (PMC2533704; doi:10.1371/journal.pgen.1000196)
Supplement: Table S1 — Strain list. (0.04 MB DOC) [file pgen.1000196.s001.doc]

Table S1. Strain List

| Strain No. | Genotypes |
| --- | --- |
| NKY1551  MSY845/846  MSY832/833  HKY276  HKY399  HKY329  HKY525  HKY447  HKY398  HKY008  HKY458  HKY406  HKY371  HKY344  HKY491  HKY397  HKY444  HKY530  HKY007  HKY412  HKY498  HKY464  HKY534  HKY475  HKY370  HKY461  HKY501  HKY524  NHY1296  HKY490 | *MAT****a*** /α*, ho::LYS2/ ho::LYS2, lys2/ lys2, ura3/ ura3, leu2::hisG/ leu2::hisG, his4X-LEU2(BamHI)-URA3/his4B-LEU2(MluI), arg4-nsp/arg4-bgl*  NKY1551 with *trp1::hisG/ trp1::hisG*  *MAT****a*** /α*, ho::LYS2 /ho::LYS2”, lys2/ lys2, ura3/ ura3, leu2::hisG/ leu2::hisG, trp1::hisG/ trp1::hisG*  MSY845/MSY846 with *csm4::KanMX6/csm4::KanMX6*  MSY845/MSY846 with *ndj1::KanMX6/ndj1::KanMX6*  MSY845/MSY846 with *msh4::TRP1/msh4::TRP1*  MSY845/MSY846 with *spo11-Y135F::KanMX6/ spo11-Y135F::KanMX6*  MSY845/MSY846 with *dmc1::URA3/dmc1::URA3*  MSY845/MSY846 with *mms4::KanMX6/mms4::KanMX6*  MSY845/MSY846 with *exo1::LEU2/exo1::LEU2*  MSY845/MSY846 with *rad50S::URA3/rad50S::URA3*  MSY845/MSY846 with *red1::LEU2/red1::LEU2*  MSY845/MSY846 with *csm4::KanMX6/ csm4::KanMX6, mms4::KanMX6/mms4::KanMX6*  MSY845/MSY846 with *csm4::KanMX6/csm4::KanMX6, msh4::TRP1/ msh4::TRP1*  MSY845/MSY846 with *csm4::KanMX6/csm4::KanMX6, ndj1::KanMX6/ndj1::KanMX6*  MSY845/MSY846 with *csm4::KanMX6/csm4::KanMX6, spo11-Y135F::KanMX6/spo11-Y135F::KanMX6*  MSY845/MSY846 with *csm4::KanMX6/csm4::KanMX6, dmc1::URA3/dmc1::URA3*  MSY845/MSY846 with *csm4::KanMX6/csm4::KanMX6, rad50S::URA3/rad50S::URA3*  MSY845/MSY846 with *csm4::KanMX6/csm4::KanMX6,* *exo1::LEU2/exo1::LEU2*  MSY845/MSY846 with *csm4::KanMX6/csm4::KanMX6,* *red1::LEU2/red1::LEU2*  MSY845/MSY846 with *ndj1::KanMX6/ndj1::KanMX6, dmc1::URA3/dmc1::URA3*  MSY845/MSY846 with *NDJ1-3HA::KanMX6/NDJ1-3HA::KanMX6*  MSY845/MSY846 with *NDJ1-3HA::KanMX6/ NDJ1-3HA::KanMX6, csm4::KanMX6/csm4::KanMX6*  MSY832/MSY833 with Rap1-GFP::*LEU2/*Rap1-GFP::*LEU2*  MSY832/MSY833 with Rap1-GFP::*LEU2/*Rap1-GFP::*LEU2, csm4::KanMX6/csm4::KanMX6*  MSY832/MSY833 with Rap1-GFP::*LEU2/*Rap1-GFP::*LEU2, ndj1::KanMX6/ndj1::KanMX6*  MSY845/MSY846 with *MPS3-3HA::KanMX6/MPS3-3HA::KanMX6*  MSY845/MSY846 with *MPS3-3HA::KanMX6/MPS3-3HA::KanMX6, csm4::KanMX6/csm4::KanMX6*  *MAT****a****/MATα, HIS4::LEU2-(BamHI)/his4-X::LEU2-(NgoMIV), leu2::hisG/leu2::hisG,* *ura3∆(sma-pst)/ ura3∆(sma-pst)*  NHY1296 with *csm4::kanMX6/csm4::kanMX6* |
