# Supplementary material for: Csm4-Dependent Telomere Movement on Nuclear Envelope Promotes Meiotic Recombination
Source: PLoS Genet. 2008 Sep 26;4(9):e1000196. doi: 10.1371/journal.pgen.1000196 (PMC2533704; doi:10.1371/journal.pgen.1000196)
Supplement: Table S2 — The number of experiments. (0.04 MB DOC) [file pgen.1000196.s002.doc]

Table S2. The number of experiments

| Figures | Number of experiments |
| --- | --- |
| Figure 1B | 2 |
| Figure 1D, E, F, G | 4 |
| Figure 2A, B, C, | 4 |
| Figure 2D, E | 3 |
| Figure 3 | 2 |
| Figure 4A, B, C | 2 |
| Figure 4D, E, F, G | 4 |
| Figure 5A | 3 |
| Figure 5B | 2 |
| Figure 5C, D | 4 |
| Figure 5E, F | 2 |
| Figure 6 | 4 |
| Figure 7A, B | 3 |
| Figure 7C | 2 |
